# Maize Arabinoxylan Gels as Protein Delivery Matrices

**DOI:** 10.3390/molecules14041475

**Published:** 2009-04-08

**Authors:** Claudia M. Berlanga-Reyes, Elizabeth Carvajal-Millán, Jaime Lizardi-Mendoza, Agustin Rascón-Chu, Jorge A. Marquez-Escalante, Ana Luisa Martínez-López

**Affiliations:** 1 Laboratorio de Biopolímeros. Centro de Investigación en Alimentación y Desarrollo, Unidad Cuauhtémoc, Avenida Río Conchos s/n Parque Industrial, Ciudad Cuauhtémoc, Chihuahua, México; 2 Laboratorio de Biopolímeros. Centro de Investigación en Alimentación y Desarrollo, Unidad Hermosillo, Carretera a la Victoria Km 0.6, Hermosillo, Sonora, México

**Keywords:** Arabinoxylan gels, Diferulic acids, Triferulic acid, Oxidative cross-linking, Protein release.

## Abstract

The laccase induced gelation of maize bran arabinoxylans at 2.5% (w/v) in the presence of insulin or β-lactoglobulin at 0.1% (w/v) was investigated. Insulin and β-lacto-globulin did not modify either the gel elasticity (9 Pa) or the cross-links content (0.03 and 0.015 μg di- and triferulic acids/mg arabinoxylan, respectively). The protein release capability of the gel was also investigated. The rate of protein release from gels was dependent on the protein molecular weight. The apparent diffusion coefficient was 0.99 × 10^-7^ and 0.79 × 10^-7^ cm^2^/s for insulin (5 kDa) and β-lactoglobulin (18 kDa), respectively. The results suggest that maize bran arabinoxylan gels can be potential candidates for the controlled release of proteins.

## 1. Introduction

Polysacharide gels have been widely investigated as matrices for protein delivery [1]. The development of oral delivery systems that improve absorption of therapeutic proteins is one of the greatest challenges in the pharmaceutical field. Due to their attractive biodegradable, biocompatible, non-toxic and hydrophilic properties and their mild cross-linking conditions, natural polysaccharides such as chitosan, starch, alginate, dextran and arabinoxylan, among others, are receiving incresing attention as colloidal carriers. Arabinoxylans are non-starch polysaccharides from the cell walls of cereal endosperm. They are constituted of a linear backbone of *β*-(1→4)-linked xylopyranosyl units to which α-L-arabinofuranosyl substituents are attached through O-2 and/or O-3, some ferulic acid (FA) esterifies arabinose at the O-5-position [2]. Feruloylated arabinoxylans can form covalent gels by oxidative coupling of FA resulting in the formation of dimers [3] and trimer [4] of FA (di-FA, tri-FA) as covalent cross-linking structures. Aarabinoxylan gels could have potential applications for colon-specific protein delivery due to their macroporous structure with mesh sizes varying from 200 to 400 nm [4], their aqueous environment and their dietary fiber nature [5]. To our knowledge, protein release capability of maize arabinoxylans gels has not been reported elsewhere. In this work maize bran arabinoxylan (MBAX) gels were formed around insulin or *β*-lactoglobulin as model proteins and the effect of protein molecular weight on the gel elasticity, cross-linking content and *in vitro* protein release capability was investigated.

## 2. Results and Discussion

### 2.1. Gelation of MBAX and MBAX-protein solutions

**Figure 1:**
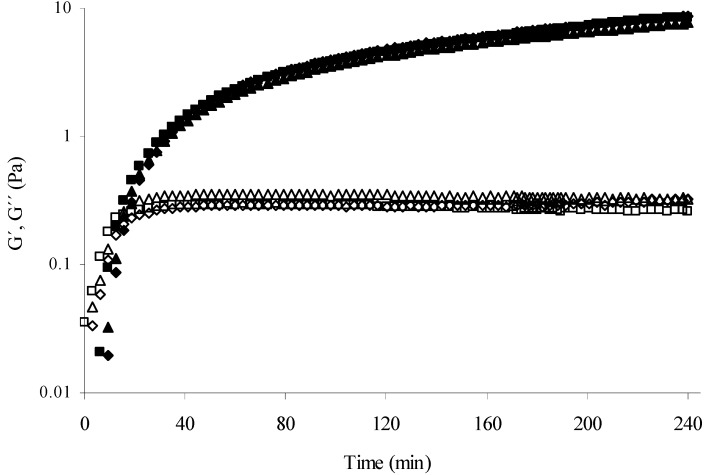
Monitoring the storage (G’) and loss (G’’) modulus of MBAX (G’ ■, G’’ □), MBAX-insulin (G’ ▲, G’’ △) and MBAX- *β*-lactoglobulin (G’ ◆, G’’ ◇) solutions during gelation by laccase at 25°C, 0.25 Hz and 5% strain. Gels at 2.5% (w/v) in MBAX and 0.1% (w/v) protein.

**Figure 2:**
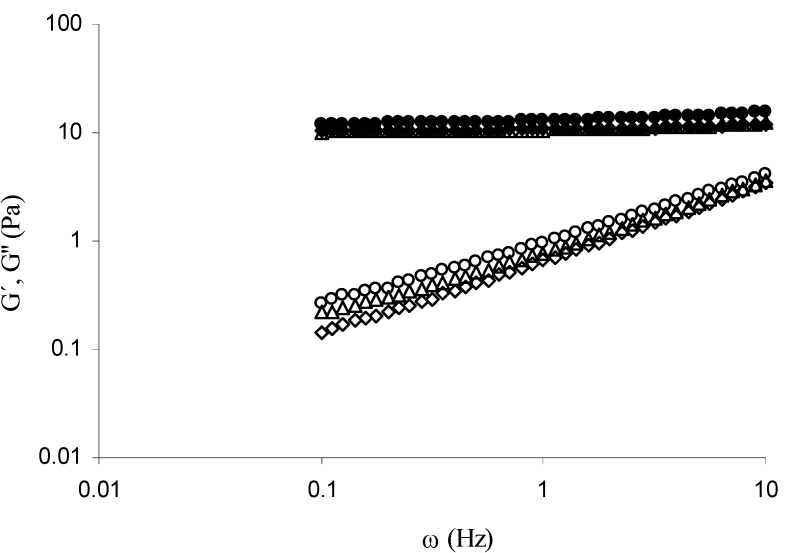
Mechanical spectrum of MBAX (G’ ■, G’’ □) and MBAX-insulin (G’ ▲, G’’ △) or MBAX-*β*-lactoglobulin (G’ ◆, G’’ ◇) gels at 4 h. Gels at 2.5% (w/v) in MBAX and 0.1% (w/v) protein. Data obtained at 25°C and 5% strain.

For all samples the storage (G´) and loss (G’’) modulus rose to reach a pseudo plateau (Figure 1). At the end of gelation (4 hours) G’ and G’’ values were 8.6 and 0.3 Pa, respectively, for MBAX gels without protein. The presence of protein did not affect significantly the G’ and G’’ final values, which varied from 7.8 to 8.8 Pa and from 0.26 to 0.32 Pa for MBAX-insulin and MBAX-β-lactoglobulin gels, respectively. In the present study 1.0 mg of insulin or β-lactoglobulin has been entrapped per mL of MBAX gel. From 1.75 to 100 mg of protein per mL of arabinoxylan gel have been entrapped in previous studies [1,6], where the addition of protein to arabinoxylan in protein/arabinoxylan mass ratio from 1 to 10 led to a 73% loss in G’ (from 41 to 11 Pa), in spite of a similar content in di-FA and tri-FA covalent cross-links. The mechanical spectra of MBAX and MBAX-protein gels after 4 h gelation (Figure 2) was typical of solid-like materials with a linear G’ independent of frequency and G’’ much smaller than G’ and dependent on frequency [7]. This behavior is similar to that previously reported for arabinoxylan gels [6]. 

At the end of gelation, 73% of the FA initially present in MBAX was oxidized, while only 12% was recovered as di and tri-FA. As a matter of fact, the di and tri-FA content in MBAX did not increase after laccase induced gelation, they rather decreased from 0.77 to 0.03 and from 0.390 to 0.015 μg/mg MBAX, respectively. This decrease in FA content without a proportional formation of di and tri-FA structures has been reported before [4,8]. These results have been related to the formation of non reported di and tri-FA or higher ferulate structures and/or to physical interactions between arabinoxylan chains. The di-FA and tri-FA content were similar in MBAX, MBAX-insulin and MBAX-β-lactoglobulin gels.

### 2.2. Controlled release of proteins

**Figure 3:**
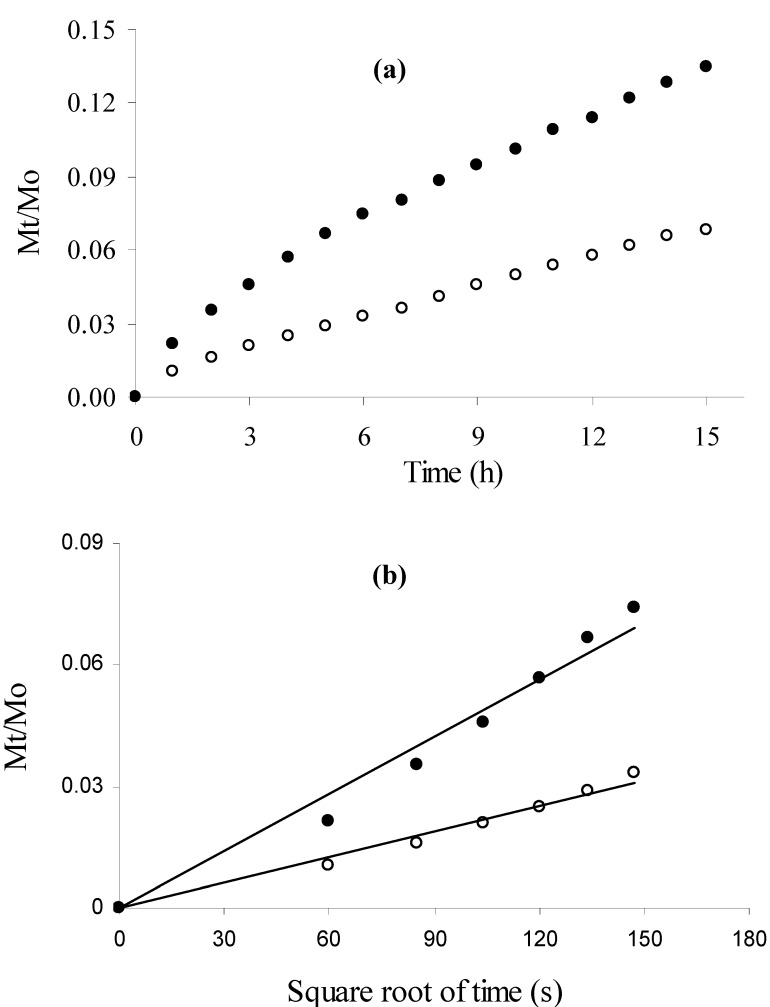
Cumulative release (Mt/Mo) of insulin (●) and *β*-lactoglobulin (○) from MBAX gels (a) as a function of time and (b) as a function of root time. Protein release was followed at 25°C and 90 rpm during 15 h. Gels at 2.5% (w/v) in MBAX and 0.1% (w/v) protein.

The kinetics of insulin or β-lactoglobulin release from MBAX gels are shown in Figure 3a. Linear relationships between Mt/Mo and the square root of time were found for insulin and β-lactoglobulin release from MBAX gels, allowing the calculation of the apparent diffusion coefficients (Dm) of these proteins in the MBAX gels (Figure 3b). 

The increase in protein molecular weight decreased the release rate. The Dm values were 0.99 × 10^-7^ and 0.79 × 10^-7^ cm^2^/s for insulin (5 kDa) and β-lactoglobulin (18 kDa), respectively (Table 1). A lower insulin Dm value (0.44-0.59 × 10^-7^ cm^2^/s) has been reported during release from temperature sensitive N-isopropylacrylamide gels [9]. Concerning β-lactoglobulin, a Dm value of 3.1 × 10^-7^ cm^2^/s was reported in polyacrylamide gel [10], which is higher to that found in the present study. The diffusion coefficient of these proteins in water (Do) obtained from the literature [11,12] are given for comparison. Do values show a difference in magnitude for both proteins, which was not reflected in the Dm values. The latter might be due to heterogeneous gel pore size and/or protein molecules distribution in the gel. Release kinetics at different concentrations of entrapped protein in MBAX gel combined with confocal microscopy studies might clarify this phenomenon. 

**Table 1:** Release parameters of insulin and *β*-lactoglobulin from MBAX gels.

Protein entrapped	Mw (kDa)	Do^a ^ × 10^-7^ (cm^2^/s)	Dm × 10^-7^(cm^2^/s)	Protein released (%)^b^
Insulin	5	15.9	0.99 ± 0.01	18 ± 1
*β*-lactoglobulin	18	7.4	0.79 ± 0.02	11 ± 1

All values are average from two repetitions: ^a^ Diffusion coefficient of proteins in water from the literature [11,12]; ^b ^ Weight of protein released after 15 h of gel incubation/weight of protein entrapped in the MBAX gel.

As presented in Table 1, the percentages of protein released by the end of the test (15 hours) were 18 and 11 % for insulin and β-lactoglobulin, respectively. These results become of importance when it is desirable to protect the carried proteins from the gastric environment for further release of functional proteins inside intestinal lumen and promote their uptake after gel degradation by colonic bacteria. Gel incubation was fixed at 15 hours as the average transit time from oral ingesta to the colonic region. Low insulin release percentages (30-45%) have been reported previously for chitosan insulin loaded gels (0.8 mg insulin/mL gel at 1% w/v chitosan), which were related to an incomplete degradation of polysaccharide gel in the *in vitro* media while *in vivo* studies reported degraded gels, allowing higher protein release [13]. 

## 3. Experimental

### 3.1. Materials

MBAX were obtained and characterized as reported before [8]. Laccase (from *Trametes versicolor*) activity was measured as previously described [4]. All chemical products were purchased from Sigma Chemical Co. (St Louis, MO, USA).

### 3.2. Methods

#### 3.2.1. Preparation of MBAX gels and MBAX-protein gels

MBAX solution at 2.5% (w/v) and MBAX-protein mixtures at 2.5 % (w/v) in MBAX and 0.1% (w/v) in insulin or β-lactoglobulin were prepared in 0.05 M citrate phosphate buffer at pH 5. Laccase (1.675 nkat/mg MBAX) was used as cross-linking agent. Gels were allowed to form for 4 h at 25°C.

#### 3.2.2. Rheological measurements

The formation of the MBAX gel was followed using a strain-controlled rheometer (AR-1500ex, TA Instruments, USA) in oscillatory mode as reported before [1] . MBAX gelation was studied for 4 h at 25°C. MBAX solution of 2.5% (w/v) MBAX were mixed with laccase (1.675 nkat per mg MBAX) and immediately placed in the cone and plate geometry (5.0 cm in diameter, 0.04 rad in cone angle) maintained at 25 °C. Exposed edges of the sample were covered with mineral oil fluid to prevent evaporation during measurements. MBAX gelation kinetics was started monitored at 25 °C for 4 h by following the storage (G’) and loss (G’’) modulus. All measurements were carried out at 0.25 Hz and 5% strain. From strain sweep tests, MBAX gels showed a linear behaviour from 1.5 to 10 % strain. The mechanical spectra of gels were obtained by frequency sweep from 0.1 to 10 Hz at 5 % strain and 25 °C. 

#### 3.2.2. Phenolic acids content

FA, di-FA and tri-FA contents in MBAX gels were quantified by reverse phase high performance liquid chromatography (RP-HPLC) after a deesterification step, as described elsewhere [1,14]. A Supelcosil LC-18-DB (250 × 4.6 mm) (Supelco, Inc., Bellefont, PA, USA) column was used. Detection was by UV absorbance at 320 nm. Isocratic elution was performed using methanol/water/acetic acid (40/59/01) at 0.6 mL/min at 35°C. A Varian 9012 photodiode array detector (Varian, St. Helens, Australia) was used to record the ferulic acid spectra. A Star Chromatography Workstation system control version 5.50 was used.

#### 3.2.3. Protein release

MBAX-insulin and MBAX-β-lactoglobulin mixtures (2 mL) were poured into a 30 mL cylindrical plastic cell (diameter 30 mm) just after laccase addition (1.675 nkat/mg MBAX). MBAX-protein gels were allowed to form for 4 h at 25°C. Then, protein was released in 0.02% (w/v) sodium azide solution (9 mL) placed on the gel surface. Gels were incubated at 25°C and 90 rpm tangential rotation and liquid medium was renewed every hour from 1 to 15 h. At the end of the test, the MBAX gels were hydrolyzed as described before [6] in order to quantify un-released protein. Protein recovery (released protein + un-released protein) was near to 100%. The protein was quantified by using the Bradford assay [15]. Protein release from MBAX-insulin and MBAX-*β*-lactoglobulin gels was characterized by calculating an apparent diffusion coefficient (Dm). This Dm was estimated from the release kinetics curve, fitted by using an analytical solution of the second Fick’s law [16], which gives the solute concentration variation as a function of time and distance:

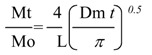

where Mt is the accumulated mass of protein released at time *t*, Mo is the mass of protein in the gel at time zero, L is the sample thickness (0.4 cm) and Dm is the diffusion coefficient. By plotting the relative solute mass released (Mt/M0) at time t versus the square root of time, a simplified determination of Dm can be made assuming that Dm is constant and that the sample is a plate with a thickness L. In this study, the apparent Dm was calculated from the linear part of the Mt/Mo (t) curves [17]. The percentage of protein released at the end of the test was also calculated. Gel incubation was fixed at 15 hours as the average transit time from oral ingesta to the colonic region.

### 3.3. Statistical analysis

All measurements were made in triplicates and the coefficients of variation were lower than 8%. Results are expressed as mean values.

## 4. Conclusions

Insulin or *β*-lactoglobulin added to MBAX solution did not affect MBAX laccase induced gelation. The cross-linking method used allowed the formation of MBAX gel in the presence of insulin or *β*-lactoglobulin without modifying the rheological properties of the gel. The protein release rate and quantity is dependent on protein molecular weight. A low amount of the entrapped protein is released by diffusion; in turn, most of the protein would be liberated only after gel degradation by colonic bacteria. These results indicate that MBAX gels could be matrices suited for protein delivery in specific sites as the colon. Additional studies will also be required in order to understand the effect of different protein concentrations in the diffusion coefficient value. 
